# Malignant Peritoneal Mesothelioma Mimicking Recurrent Diverticulitis

**DOI:** 10.7759/cureus.3906

**Published:** 2019-01-17

**Authors:** Ernesto S Robalino Gonzaga, Patricia Guzman Rojas, Vishwas Vanar

**Affiliations:** 1 Internal Medicine, University of Central Florida College of Medicine, Orlando, USA

**Keywords:** mesothelioma, diverticulitis

## Abstract

Mesothelioma is an uncommon type of cancer arising from the mesothelial cells that form the lining of several cavities in the body. Exposure to asbestos is the leading known cause of mesothelioma.

We present a 73-year-old male with a significant asbestos exposure and a recent history of recurrent diverticulitis who reported persistent left lower quadrant (LLQ) pain despite several courses of empiric antibiotic therapy. A recent computed tomography (CT) performed due to nonresolving symptoms showed possible nodularity of the mesentery and subsequent positron emission tomography (PET) scan demonstrated multiple hypermetabolic mesenteric lesions, notably in the left paracolic gutter. A colonoscopy was subsequently performed which demonstrated severe diverticulosis, but no obvious luminal lesions. The patient underwent an exploratory laparoscopy showing extensive peritoneal carcinomatosis involving all mesenteric surfaces and partial involvement of the right diaphragm. Final pathology revealed malignant epithelial mesothelioma with peritoneal seeding. The patient was referred to oncology and was started on hyperthermic intraperitoneal chemotherapy (HIPEC) and cytoreductive surgery (CRS).

Our case highlights a challenging presentation of malignant peritoneal mesothelioma (MPM), which is often initially misdiagnosed due to vague symptoms. Physicians should consider further diagnostic workup for unrelenting LLQ abdominal pain after diverticulitis has been treated.

## Introduction

Mesothelioma is an uncommon cancer of the mesothelial cells that form the lining of the pleural, peritoneal, pericardial, and reproductive organs. Mesothelioma arises most frequently from the pleura (65%-70%), followed by the peritoneum (10%-15%), then tunica vaginalis testis, and the pericardium (1%-2%). Incidence rates in industrialized countries range between 0.5 and three cases per million in men, and between 0.2 and two cases per million in women [1].

Approximately 3,300 cases of malignant mesotheliomas are diagnosed every year in the USA, with 10%–15% of this number representing malignant peritoneal mesothelioma (MPM) [2].

Here, we report a rare presentation of MPM. This is the first case report in the literature that elucidates this atypical presentation.

## Case presentation

**Figure 1:**
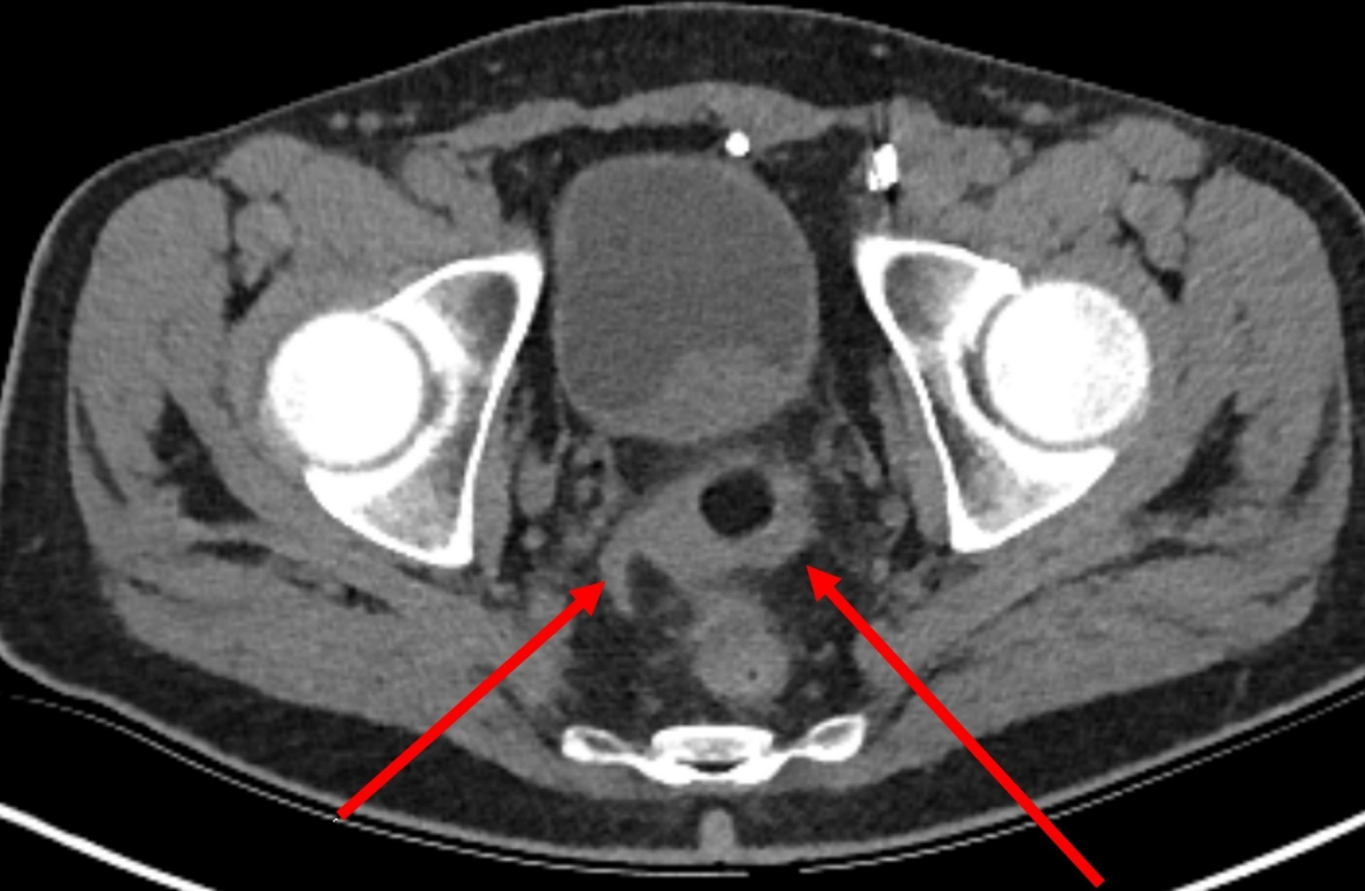
Computed tomography (CT) scan of the abdomen showing mild inflammation/fluid adjacent to distal sigmoid colon.

**Figure 2:**
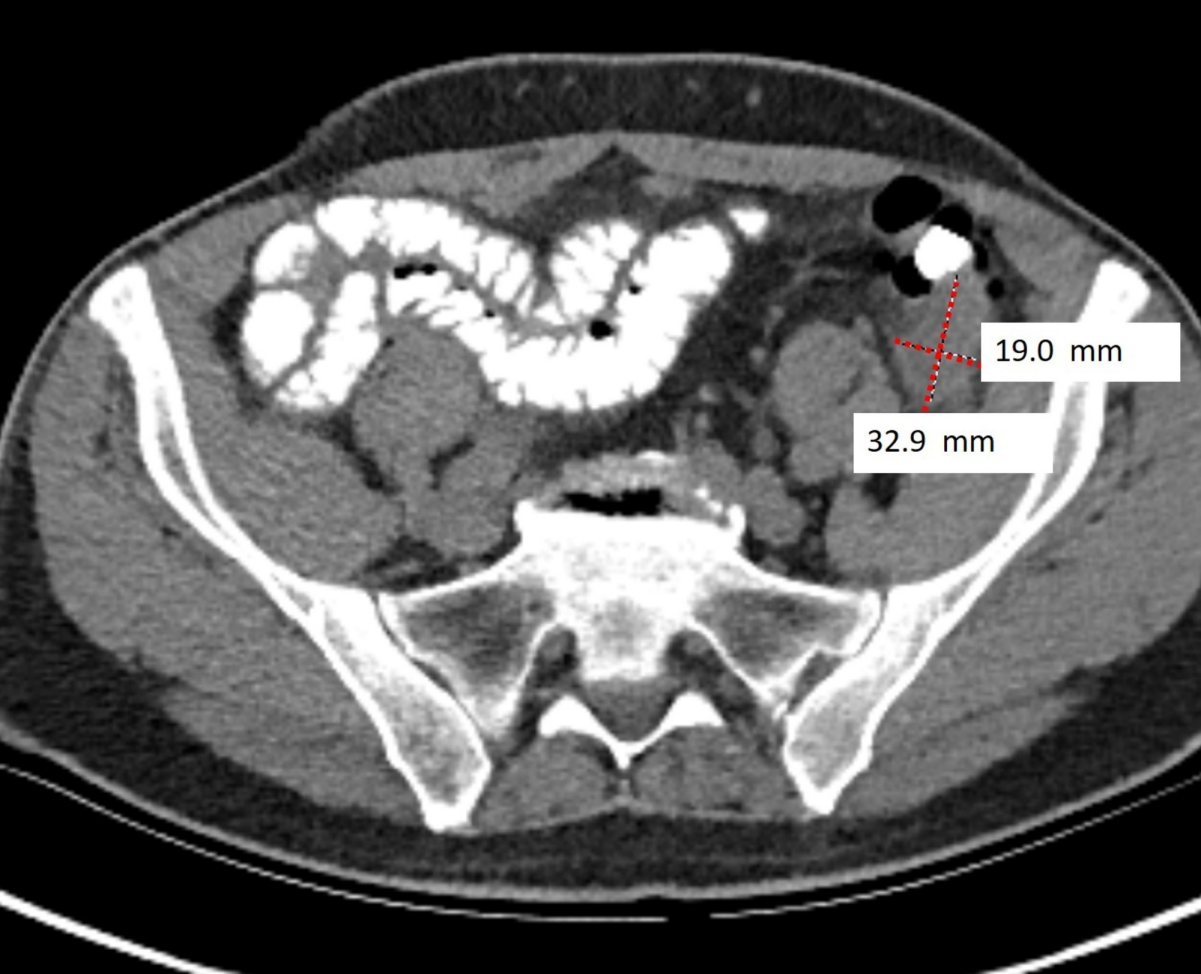
Repeat CT scan showing a peritoneal mass, thought to be postinflammatory process (diverticulitis).

**Figure 3:**
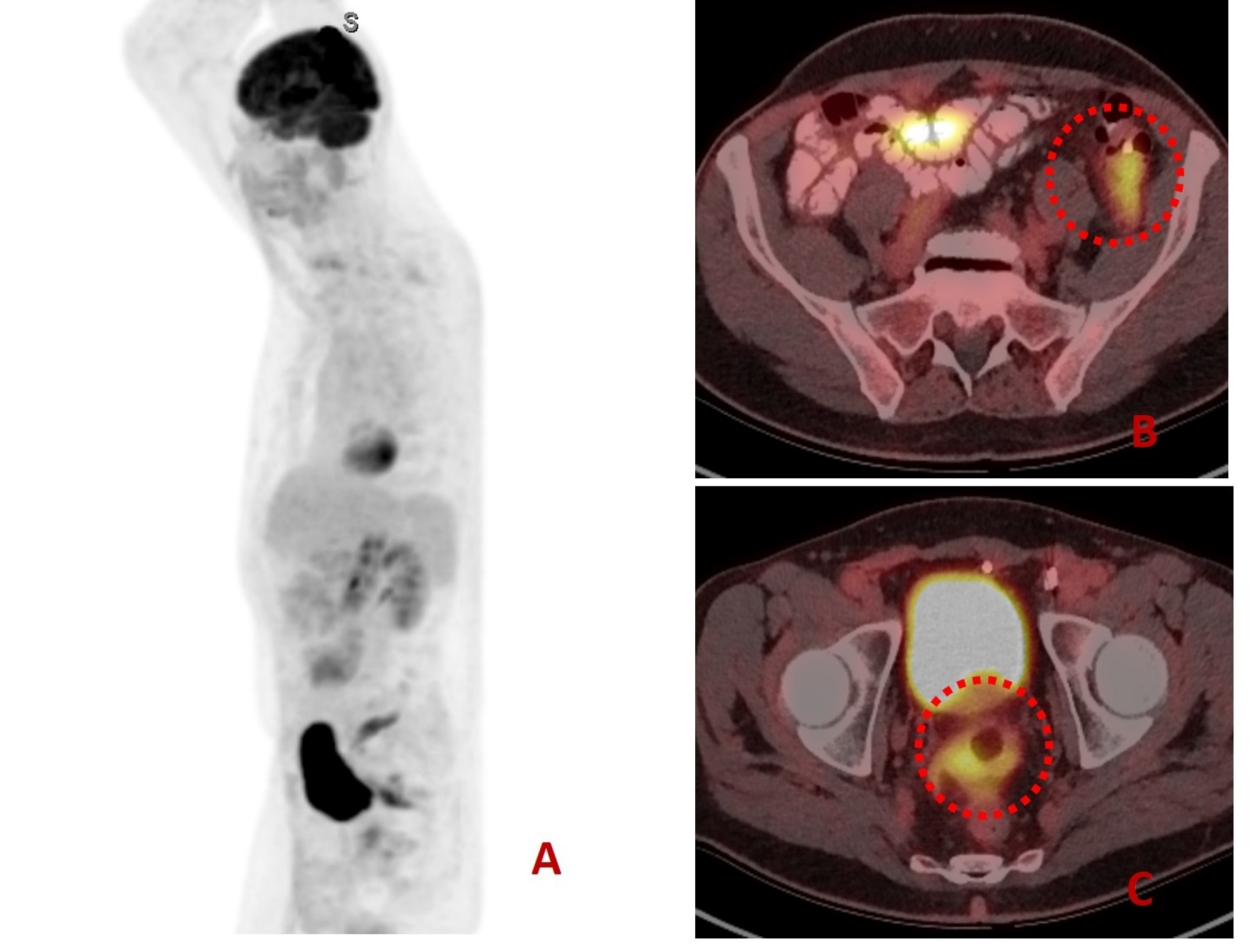
Positron emission tomography (PET) showing whole body and axial views. A:  Whole body image showing focal, intensely increased activity in the pelvic region. B: On axial image, there is increased activity (red circle) at left paracolic gutter. C: On axial image, there is increased activity at pelvic area (red circle).

A 73-year-old male with a significant asbestos exposure and a recent history of recurrent diverticulitis presented to the gastroenterology clinic with persistent left lower quadrant (LLQ) pain despite several courses of empiric antibiotic therapy. Computed tomography (CT) scan completed during a previous hospitalization which showed fluid near sigmoid colon suggested nonspecific colitis (Figure 1). Repeat CT performed due to nonresolving symptoms showed possible nodularity of the mesentery (Figure 2). Subsequent positron emission tomography (PET) scan demonstrated multiple hypermetabolic mesenteric lesions, notably in the left paracolic gutter and portion of pelvis (Figure 3). The findings favored carcinomatosis. A colonoscopy was subsequently performed which demonstrated severe diverticulosis, but no obvious luminal lesions. Tumor marker serology was negative. The patient was eventually referred to colorectal surgery and an exploratory laparoscopy was done. He was noted to have extensive peritoneal carcinomatosis involving all mesenteric surfaces and partial involvement of the right diaphragm. The disease involved predominantly the LLQ, with encasement of the left colon over the pelvic brim and into the pelvis between the bladder and colon. A sigmoid colon resection with diverting colostomy was performed to provide symptomatic relief. Final pathology revealed malignant epithelial mesothelioma with peritoneal seeding. The patient was referred to oncology and was started on hyperthermic intraperitoneal chemotherapy (HIPEC) and cytoreductive surgery (CRS).

## Discussion

Exposure to asbestos is the leading known cause of pleural mesothelioma; however, the association is weaker in cases of peritoneal mesothelioma. Other less common causes of mesothelioma include exposure to ionizing radiation, chronic pancreatitis, and genetic factors; however, there is no strong evidence that confirms these exposures as risk factors [3]. Our patient is a navy veteran who worked in a ship for many years during the 1960s. For this reason, we believed that asbestos was the main factor for which he developed the peritoneal mesothelioma.

The most frequently reported initial symptoms are abdominal pain (35%), abdominal swelling (31%), anorexia, marked weight loss, and ascites. Night sweats and hypercoagulability have been reported less frequently [1]. Clinical signs that are common to mesothelioma include fever of unknown origin, intestinal obstruction, and/or surgical/acute abdomen due to acute inflammatory lesions. Late (advanced stage) complications include external compression or obstruction of the gastrointestinal tract [4]. As abdominal pain is the most common initial symptom, patients usually undergo laboratory testing and abdominal plain film or CT scan. Routine laboratory tests are not useful for diagnosis, and similarly plain films only show nonspecific obstructive patterns of abdominal distention and are not useful in identifying the root cause. The CT findings may show peritoneal thickening and irregularity and/or a nodular pattern, soft tissue mass in the abdominal or pelvic cavity, and/or bowel wall thickening. Serum mesothelin-related protein (SMRP) is a commonly elevated tumor marker found in more than 84% of mesotheliomas, and has a 60% sensitivity at diagnosis [5]. CA-125, CA 15-3, hyaluronic acid, and osteopontin are other potential markers [6].

Ascites and pleural disease (such as thickening, calcification, and effusion) can be common signs of advanced stage. Metastasis to the liver, lung or bone, along with retroperitoneal lymphadenopathy, and calcification has been reported to be relatively uncommon feature in mesothelioma [6]. Cytologic analysis of peritoneal ascites or pleural effusion has a low diagnostic potential due to high cytologic diversity of tumor cells and a small number of malignant cells within the fluid. When no effusion is present, sampling by fine-needle aspiration of a mass can be used to reach a diagnosis. Ultimately, biopsy is the gold standard for diagnosis [7].

Standard treatment options include CRS along with HIPEC. Both treatments have shown the most promising outcomes in improving survival outcomes and offering palliative measures for advanced disease. Specifically, HIPEC with the use of cisplatin chemotherapy has shown to be independently more successful with survival rates versus HIPEC with mitomycin-c chemotherapy [8].

Prognosis for mesothelioma after diagnosis is often poor. The median survival in patients undergoing any intervention is approximately two years [9-10]. Factors associated with longer survival include young age, complete or near complete gross tumor resection, and histologic tumor grade. The operative mortality is approximately 2.3% [7], and the overall median survival for patients following CRS and HIPEC ranges from 34.2 months to 92 months. Patients who do not receive any treatment have a median survival of six months [8].

## Conclusions

Our case highlights a challenging presentation of MPM. Diverticulitis is a common entity that has been rising in the last decade mainly in the elderly population and can be mistakenly over-diagnosed. Accurate history taking may identify possible occupational exposures which can expand our differential diagnosis. Physicians should consider further diagnostic workup for unrelenting LLQ abdominal pain after diverticulitis has been treated. This simple step can help shorten the delay in diagnosis and ultimately treatment.
